# Oxidative MLD of Conductive PEDOT Thin Films with EDOT and ReCl_5_ as Precursors

**DOI:** 10.1021/acsomega.1c02029

**Published:** 2021-07-01

**Authors:** Saba Ghafourisaleh, Georgi Popov, Markku Leskelä, Matti Putkonen, Mikko Ritala

**Affiliations:** Department of Chemistry, University of Helsinki, P.O. Box 55, Helsinki FI-00014, Finland

## Abstract

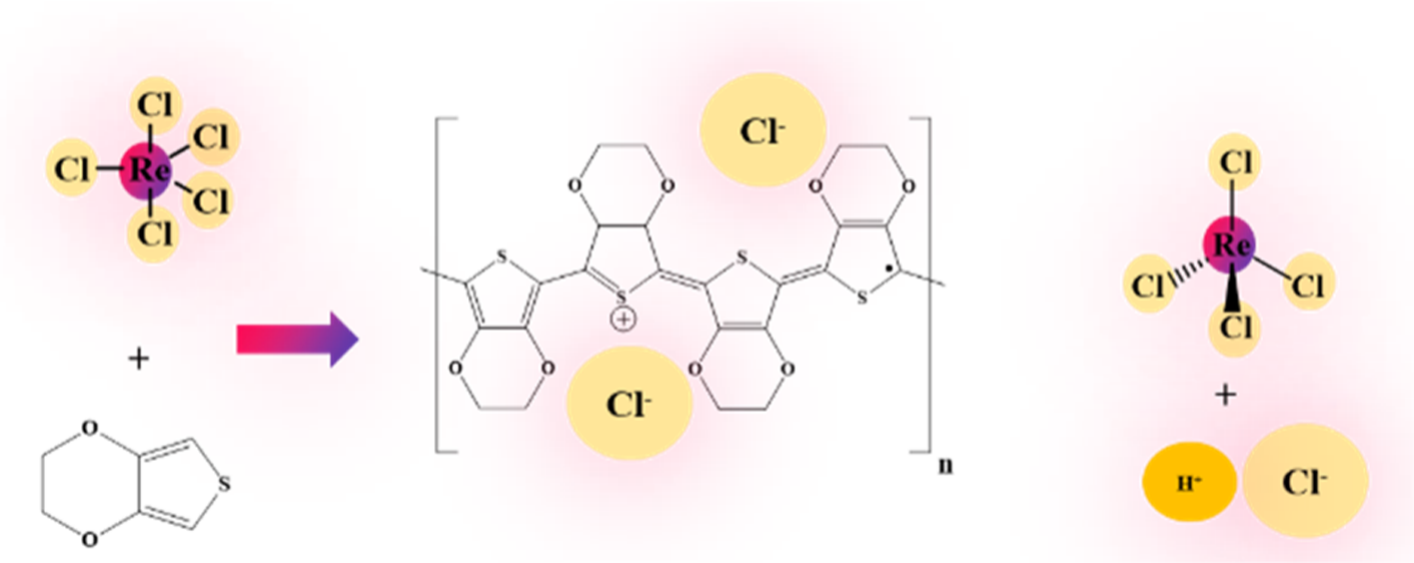

Because of its high conductivity and intrinsic stability, poly(3,4-ethylenedioxythiophene (PEDOT) has gained great attention both in academic research and industry over the years. In this study, we used the oxidative molecular layer deposition (oMLD) technique to deposit PEDOT from 3,4-ethylenedioxythiophene (EDOT) and a new inorganic oxidizing agent, rhenium pentachloride (ReCl_5_). We extensively characterized the properties of the films by scanning electron microscopy, X-ray diffraction, X-ray photoelectron spectroscopy (XPS), energy-dispersive X-ray spectroscopy (EDS), Raman, and conductivity measurements. The oMLD of polymers is based on the sequential adsorption of the monomer and its oxidation-induced polymerization. However, oMLD has been scarcely used because of the challenge of finding a suitable combination of volatile, reactive, and stable organic monomers applicable at high temperatures. ReCl_5_ showed promising properties in oMLD because it has high thermal stability and high oxidizing ability for EDOT. PEDOT films were deposited at temperatures of 125–200 °C. EDS and XPS measurements showed that the as-deposited films contained residues of rhenium and chlorine, which could be removed by rinsing the films with deionized water. The polymer films were transparent in the visible region and showed relatively high electrical conductivities within the 2–2000 S cm^–1^ range.

## 1 Introduction

Poly(3,4-ethylenedioxythiophene) (PEDOT) is one of the best-known conjugated conductive polymers that has been extensively studied for decades.^1^ Other well-known conjugated conductive polymers, polypyrrole and polyacetylene, were discovered earlier in the pioneering work of Shirakawa et al.^2−4^

Compared to the other conductive polymers, PEDOT has attracted lots of attention because of its high and stable electrical conductivity. Electrical conductivities up to 8797 S cm^–1^ and 7520 S cm^–1^ have been reported for single crystals^5^ and thin films,^6^ respectively. These conductivity values are just 1 order of magnitude lower than those of the most conductive metals such as silver and copper.^7^ Conductive polymers are often highlighted for their mechanical, electrical, optoelectronic, thermoelectric, photovoltaic, and lighting properties both academically and industrially. The significant importance of PEDOT is acknowledged through the high number of applications in thermoelectricity, photovoltaics, lighting, sensing, technical coatings, transparent electrodes, bioelectronics, and so forth.^8−14^

Although there are a myriad of publications dealing with the synthesis, properties, conductivity enhancement, and different applications of PEDOT, there is still room for a deeper understanding of the materials’ properties, whereas novel deposition methods can enable its use in new applications. PEDOT thin films can be deposited by both liquid- and gas-phase methods. In situ chemical polymerization (ICP)^15−19^ was the first and widely utilized film-forming method that electrochemically synthesizes PEDOT. Oxidative ICP was carried out in EDOT solutions with chemical oxidants such as metal salts, peroxides, and other more sophisticated oxidants.^8^ Typically, ICP of PEDOT results in high conductivity, poor transparency, and small sample sizes.^20^ Another promising approach is based on spin-casting of the 3,4-ethylenedioxythiophene (EDOT) monomer and oxidizing agents onto a variety of different substrates to oxidatively polymerize the monomers. Films with high transparency and conductivity values of 300 S cm^–1^ have been reported.^21^ PEDOT and other conductive polymer films have also been electrodeposited for different applications.^22,23^ For instance, electrodeposition of polyaniline (PANI)-PEDOT on an indium tin oxide substrate with gold nanostructures was reported by Popov et al.^24^ as an example of enhancing the electrochromic properties of conductive polymers. Moreover, a comparison study on the electrical conductivity of the electrodeposited polymer films such as PEDOT, PANI, and nanocomposite of PANI-PEDOT has been reported.^25^

Vapor-phase polymerization (VPP)^20,26−28^ is based on a similar concept to ICP, but instead of mixing EDOT to the oxidant solution, oxidants adsorbed on a substrate surface are exposed to EDOT vapors.^20^ A recent approach to the PEDOT film deposition is chemical vapor deposition (CVD)^29−34^ using EDOT and FeCl_3_,^5,35^ Br_2_,^36^ MoCl_5_,^37^ SbCl_5_,^38^ or VOCl_3_^39^ as an oxidant. Both gas-phase techniques, VPP and CVD, rely on the oxidation of the EDOT monomer by an oxidant, and a similar approach has been used also with oxidative molecular layer deposition (oMLD).^37^ The principle of MLD^40−42^ is stemming from the atomic layer deposition (ALD) technique.^43,44^ In both methods, film growth occurs through self-limiting surface reactions between alternately supplied precursor vapors. The self-limiting film growth mechanism gives ALD and MLD superior characteristics as compared to the competing methods: uniform and conformal films over also large and three-dimensionally structured substrates, and accurate and simple film thickness control. Previously, Atanasov et al.^37^ used MoCl_5_ as an oxidant together with the EDOT monomer for the oMLD of PEDOT thin films. The films were deposited at temperatures of 100–150 °C. According to X-ray photoelectron spectroscopy (XPS) analysis, approximately 6 at. % residuals from MoCl_5_ with different oxidation states of Mo were present in the films exposed to ambient air after the deposition. In an attempt to improve the film purity, we studied PEDOT thin film deposition by the oMLD technique using alternative oxidizers. Inspired by the MoCl_5_-based process, we explored oxidizers, which have been previously used for oxidation of thiophene monomer derivatives in CVD such as Br_2_, SbCl_5_, and VOCl_3_.^36,38,39^ Rhenium pentachloride (ReCl_5_) was identified as another potential oxidizer based on its similar properties and structure to MoCl_5_. To the best of our knowledge, there are no previous reports on using ReCl_5_ as an oxidizer.

## 2 Results and Discussion

### 2.1 PEDOT Film Growth

In the initial experiments besides ReCl_5_, bromine, antimony pentachloride, vanadium oxytrichloride, and ozone were used as oxidizers, but only ReCl_5_ led to PEDOT deposition.

**Figure 1:**
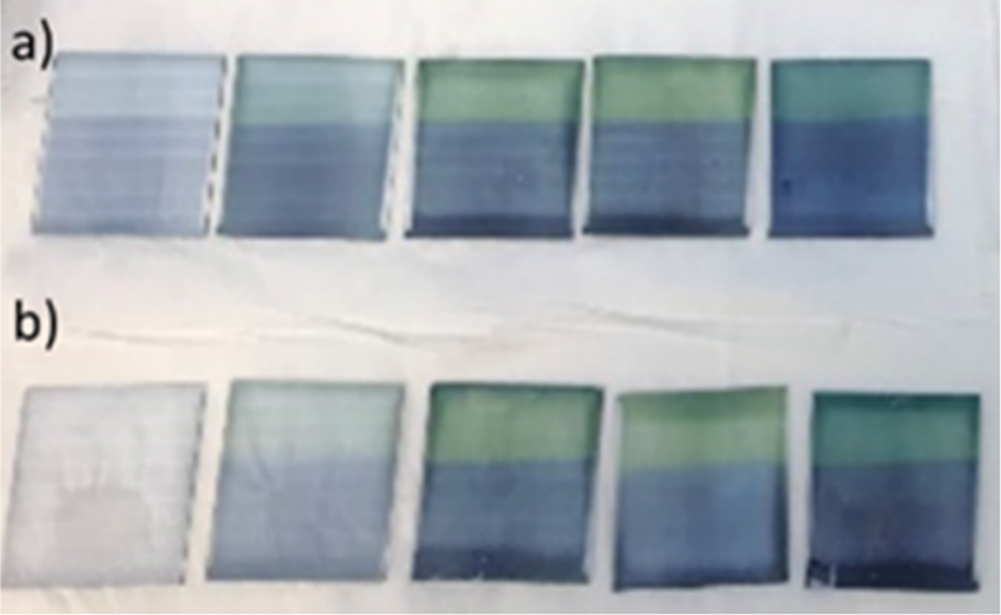
Photograph of the films deposited at 200 °C with 1000 cycles on soda-lime glass substrates and dipped halfway in water (lower half), using (0.2, 0.5, 1.0, 2.5, and 3.0 s) pulses. (a) ReCl_5_ and (b) EDOT.

Atanasov et al.^37^ demonstrated the deposition of PEDOT thin films in the 100–150 °C temperature range with MoCl_5_. ReCl_5_ is structurally and chemically similar to MoCl_5_, with a similar oxidation potential for the EDOT oxidation. In our present study, somewhat higher deposition temperatures were needed because the source temperature of ReCl_5_ was 110–115 °C. We were able to deposit PEDOT thin films in the temperature range of 125–200 °C. Films deposited at 200 °C were uniform, reflective on silicon, and transparent on soda-lime glass (Figure 1). Increasing the temperature to above 200 °C decreased the growth rate dramatically and no film was achieved at temperatures higher than 200 °C. Deposition temperatures lower than 200 °C had a considerable effect on the film quality and appearance. Films deposited at these temperatures were flaky, nonuniform, and opaque black. Therefore, 200 °C was selected as a temperature to deposit PEDOT films for further analysis.

**Figure 2:**
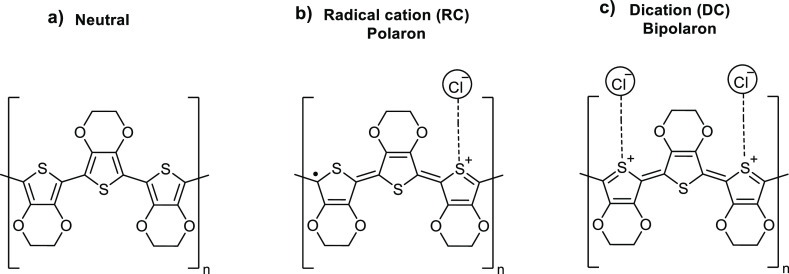
PEDOT oligomer chains with three molecular structures. (a) Neutral, (b) RC or polaron, and (c) DC or bipolaron structure.

The effect of precursor pulsing times on the deposition process was studied at 200 °C. With both precursors, no full saturation of the growth rate was observed (Figure 2). This was unexpected because EDOT and ReCl_5_ are reported to be stable at the temperatures in question.^45^

**Scheme 1:**
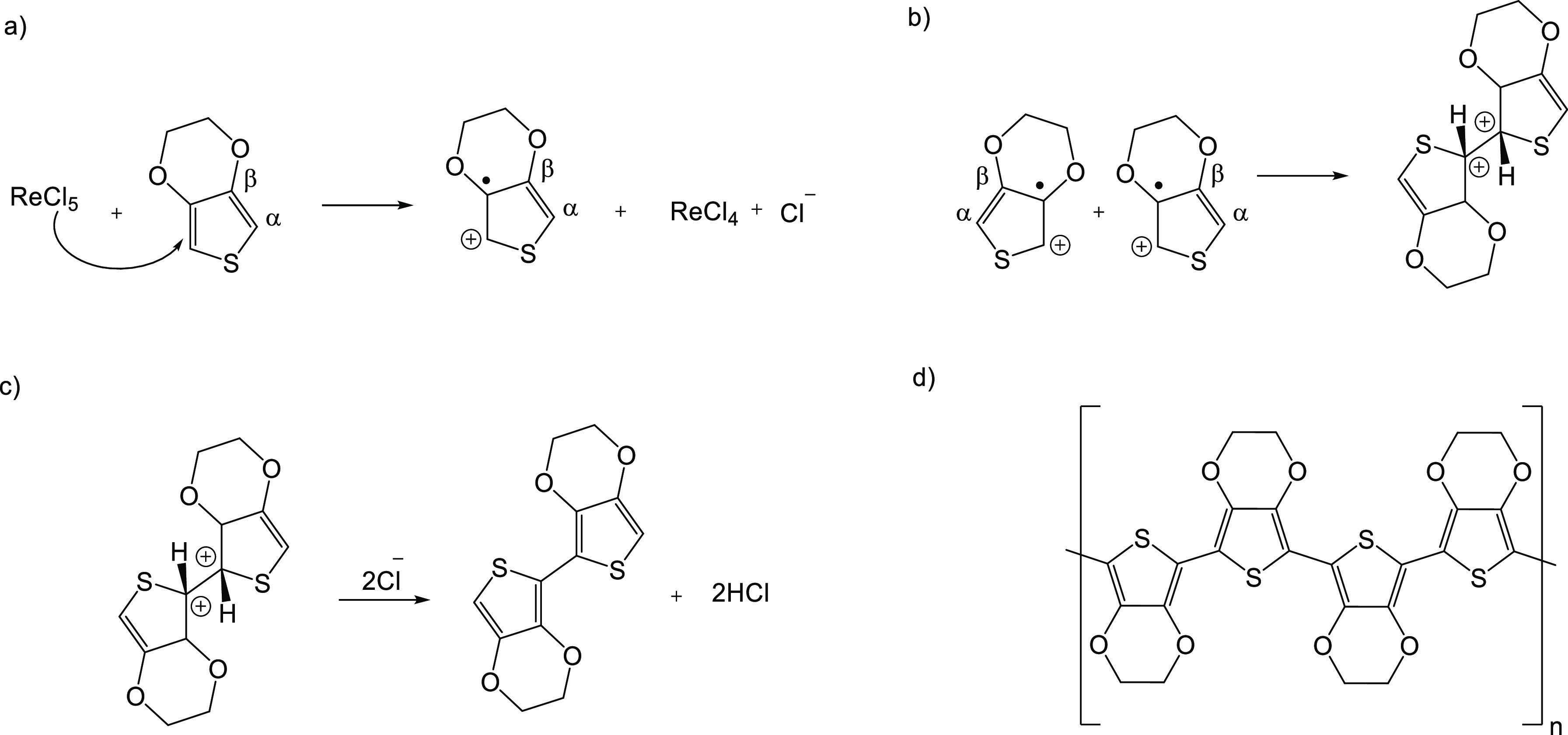
Possible Mechanism for the Polymerization of the EDOT by ReCl_5_. (a) Dimerization, (b) stabilization by Chloride Anions, (c) removal of the Protons, and (d) Polymerization

The PEDOT polymer is assumed to be deposited through a self-limiting radical cation (RC) transition mechanism.^46−48^ During the ReCl_5_ pulse, a monolayer of ReCl_5_ is chemisorbed on the surface. During the EDOT pulse, ReCl_5_ oxidizes EDOT and transforms it into a RC (Scheme 1a) that dimerizes (Scheme 1b) and is rapidly stabilized through removal of the two protons by chloride anions as counter ions and dopants (Scheme 1c). Additional ReCl_5_ oxidizes the dimers and the chain growth continues as a classical step-polymerization forming the PEDOT polymer (Scheme 1d). Once all ReCl_5_ is consumed from the surface, the growth stops. The next ReCl_5_ pulse adds a new layer of ReCl_5_ molecules on the surface to continue the growth. ReCl_5_ also oxidizes the PEDOT chains into a doped (conducting) state. Some of the chloride anions released through this process can get trapped in the polymer media and act as counter ions for the polymer cations to stabilize the polymer structure. These interactions can also enhance the electrical conductivity properties of the polymer. Polymer is self-doped after the polymerization and can efficiently conduct electricity as will be seen later.

As the polymerization continues, the oligomeric PEDOT chains can appear in three molecular structures; neutral, RC, and dication (DC) (Figure 2). Because PEDOT is a conjugated conductive polymer, the charge carriers created by doping with chloride ions are in nondegenerate energy levels consisting of polarons (RC) and bipolarons (DC).

As discussed in more detail later, according to energy-dispersive X-ray spectroscopy (EDS), the film surfaces had Re- and chlorine-containing particulates originating from ReCl_5_. As PEDOT is insoluble in water, rinsing with water was tested and indeed the particulates could be easily removed by rinsing the films with deionized water at 25 °C.

On glass, the films were clearly transparent. The as-deposited films were gray-green due to the residual impurities. The films became blue and maintained their transparency and uniformity after the rinsing (Figure 1). PEDOT in its natural doped state has a sky-blue color. There are various factors that can change the color of the polymer.^9^

**Figure 3:**
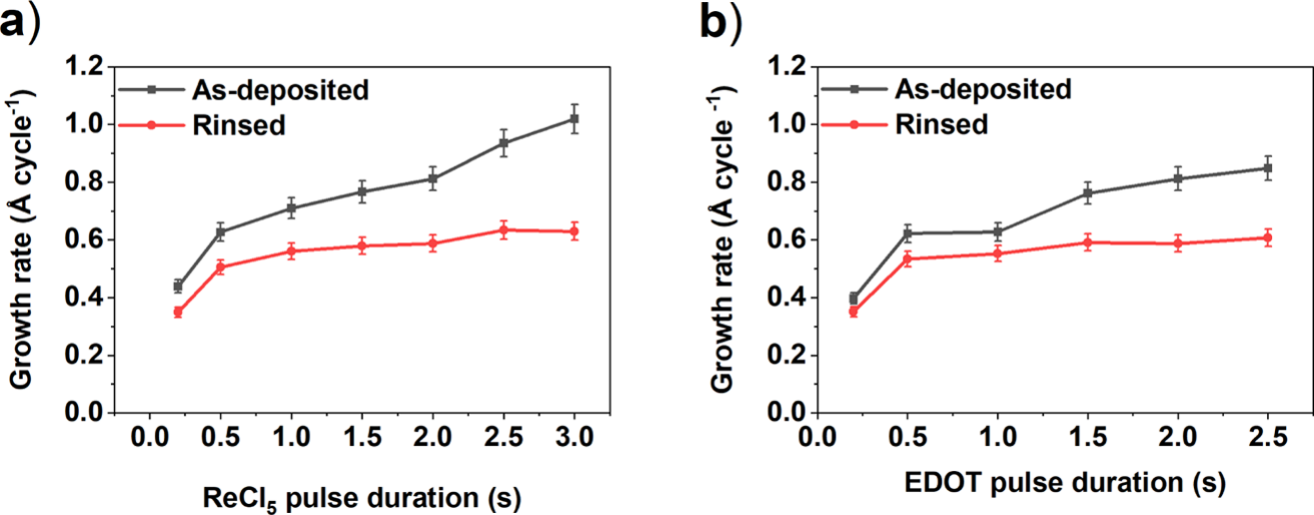
Growth rates of the films deposited at 200 °C by varying the pulsing durations of (a) ReCl_5_ and (b) EDOT.

The water rinsing after the deposition caused a 20% thickness decrease (from 63–71 to 51–56 nm) for the films deposited with the shortest pulsing times of 0.5–1.0 s, whereas the films deposited with a 2.0 s ReCl_5_ pulse time encountered a 27% thickness decrease (from 81 to 59 nm). Once the growth rates were evaluated from the rinsed thin films, they showed better saturation and remained relatively constant around 0.5 Å cycle^–1^ when the ReCl_5_ or EDOT pulse times were increased (Figure 3).

### 2.2 Electrical and Optical Properties

**Figure 4:**
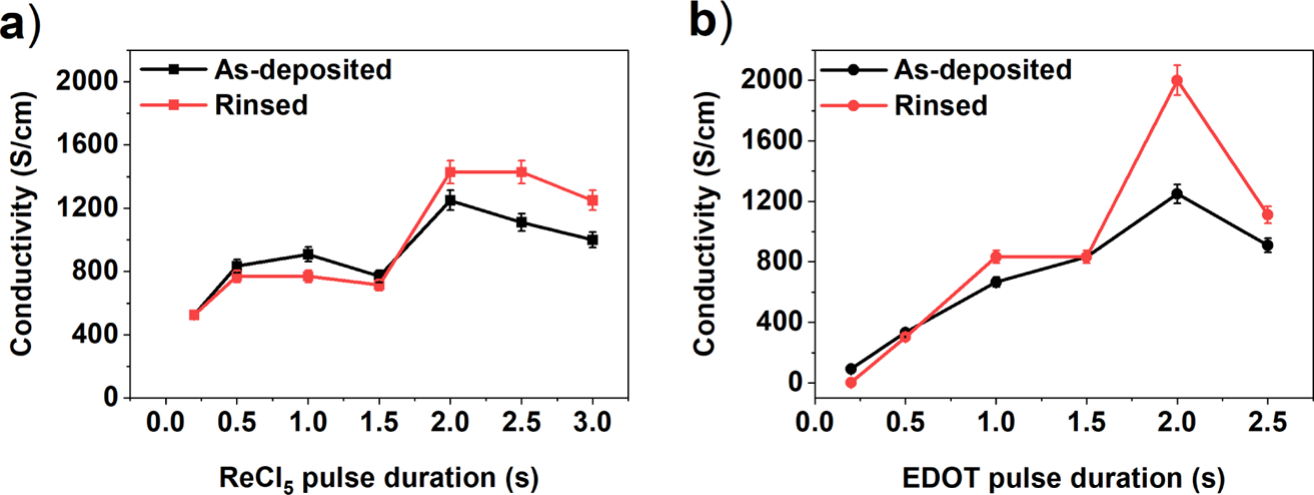
Conductivity of the as-deposited and water-rinsed samples. Effect of (a) ReCl_5_ pulse duration and (b) EDOT pulse duration.

We studied the electrical conductivity of our films by measuring the sheet resistance with a four-point probe. In order to compare the electrical conductivity of the films before and after the postdeposition water-rinse, we dipped the films on glass substrates only halfway to water (Figure 1) and the thicknesses and electrical conductivities of the two-halves were measured and compared (Figure 4).

We measured conductivities exceeding 2000 S cm^–1^. The conductivities of the thicker films increased after the water rinsing, whereas in the thinner films the water rinsing did not cause any significant conductivity changes.

PEDOT films deposited with various deposition methods^53−57^ have had different conductivities. There have been numerous studies on enhancement of the conductivities of PEDOT thin films, where the conductivity was enhanced by dopant treatments after the deposition.^7,19,33,37,46,50^ For instance, Song et al.^53^ reported a conductivity of 2673 S cm^–1^ for flexible high-conductivity transparent electrode based on PEDOT: poly(styrene sulfonate) (PEDOT:PSS) deposited on flexible plastic substrates via a H_2_SO_4_ treatment. This was among the highest electrical conductivities of PEDOT:PSS films processed on flexible substrates. The electrodes demonstrated high transparency, over 60%, at 550 nm.

Heydari Gharahcheshmeh et al.^6^ reported an optimized electrical conductivity of 7520 ± 240 S cm^–1^, which is a record for PEDOT thin films. These films were face-on oriented semicrystalline PEDOT and were deposited by water-assisted oxidative chemical vapor deposition with antimony pentachloride as an oxidant. To the best of our knowledge, 8797 S cm^–1^ is the highest conductivity that has been measured for PEDOT single crystals.^5^

Generally, conductivity is highly affected by the orientation of semicrystalline-conjugated polymers, which is strongly influenced by the fabrication method, size of the counter-ion dopant, and process parameters.^49^ The predominant edge-on orientation is frequently reported in PEDOT:PSS, PEDOT:Tos, and PEDOT:OTf processes,^19,51,52^ while the use of smaller counter-ion dopants such as chloride (Cl^–^) induces the predominant face-on orientation in PEDOT:Cl thin films.^39,50^

Most of the conductivities reported for PEDOT thin films in their natural self-doped state have been around 1000 S cm^–1^.^7^ Compared to these values, the films deposited by our process without any extra doping treatments showed somewhat higher electrical conductivities.

**Figure 5:**
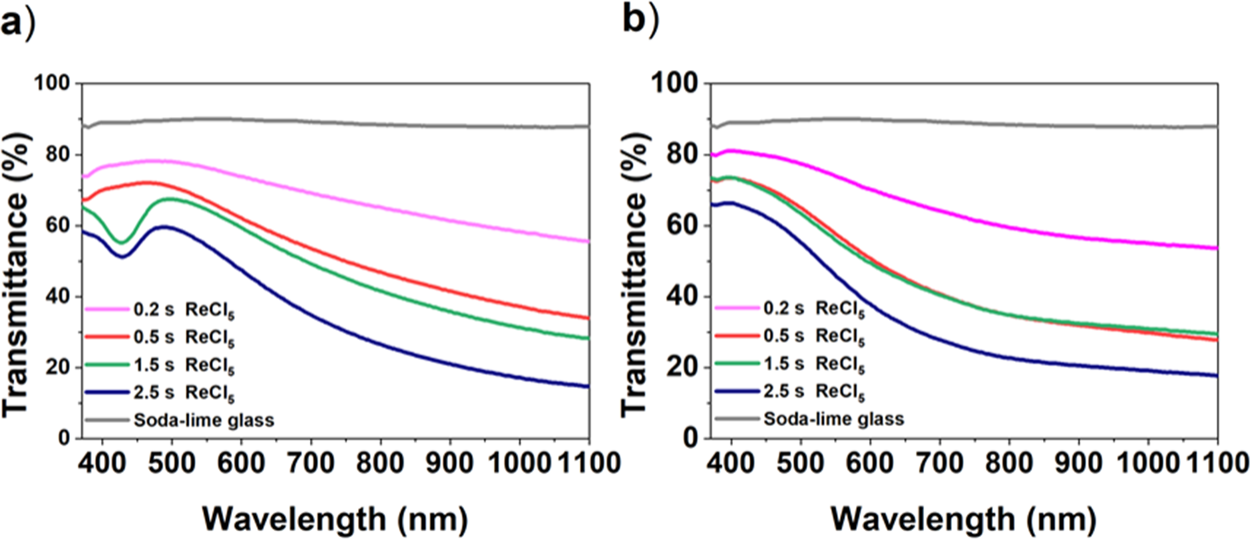
Transmittance of the (a) as-deposited films with various ReCl_5_ pulse lengths and (b) transmittance of the rinsed films.

To confirm the effect of the water rinsing on the transparency of the PEDOT films, their transmittance was measured with a UV/Vis spectrometer with a bare soda-lime glass as the reference (Figure 5). The rinsed films showed higher transmittance than the as-deposited films. For the rinsed films, the highest transmittance value was 80%, while the corresponding value for the as-deposited film was 75%.

With increasing thickness of the films, the transparency decreased. In the thicker as-deposited films, there is one dip in transmittance at 425 nm for 1.5 and 2.5 s pulses of ReCl_5_, but this dip disappeared after the water rinsing.

### 2.3 Film Structure and Composition

**Figure 6:**
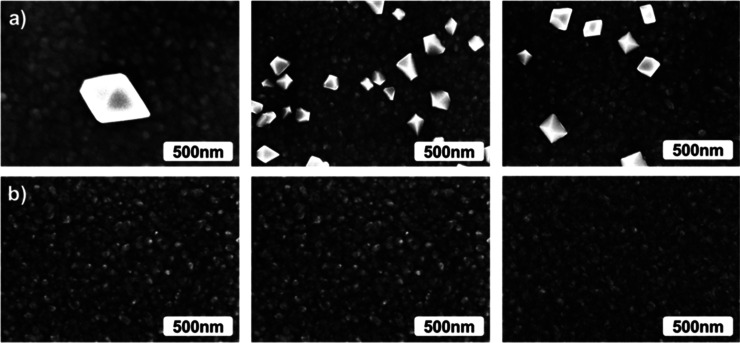
SEM images of (a) as-deposited and (b) water-rinsed PEDOT films deposited with 1000 cycles at 200 °C using, from left to right, 0.2, 0.5, and 1.0 s pulses of ReCl_5_.

Scanning electron microscopy (SEM) images showed that the as-deposited films are continuous, but segregated particulates were present on their surface (Figure 6a). EDS analysis indicated that the particulates contained rhenium and chlorine impurities. Effects of the ReCl_5_ pulse times on the film morphology can be seen in SEM images (Figure 6a). It appears that the amount of particulates increases with the increasing ReCl_5_ pulse time, thus at least partially explaining the nonsaturating growth observed in the as-deposited films while increasing precursor pulse times (Figure 3). After the water rinsing, the particulates had been removed from the film surfaces (Figure 6b). Some Re impurities were still measured with EDS but no chlorine could be detected.

**Figure 7:**
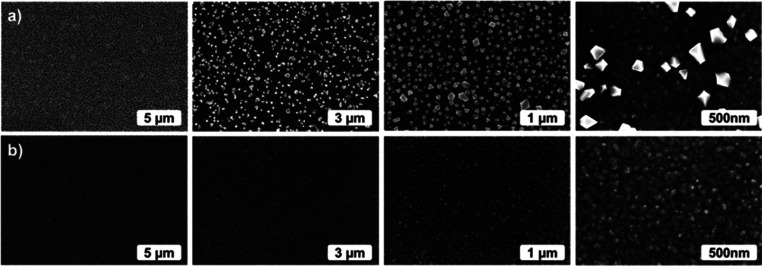
SEM images at different magnifications of (a) as-deposited and (b) water-rinsed PEDOT films deposited with 1000 cycles at 200 °C using, a 0.5 s pulse of ReCl_5_.

In order to show the coverage of the particulates on the film surface after the deposition in detail, we imaged the surface morphologies of the PEDOT film deposited with 0.5 s pulses of ReCl_5_ in different magnifications (Figure 7a). After the water rinsing, the particulates were again removed from the film surface (Figure 7b).

**Figure 8:**
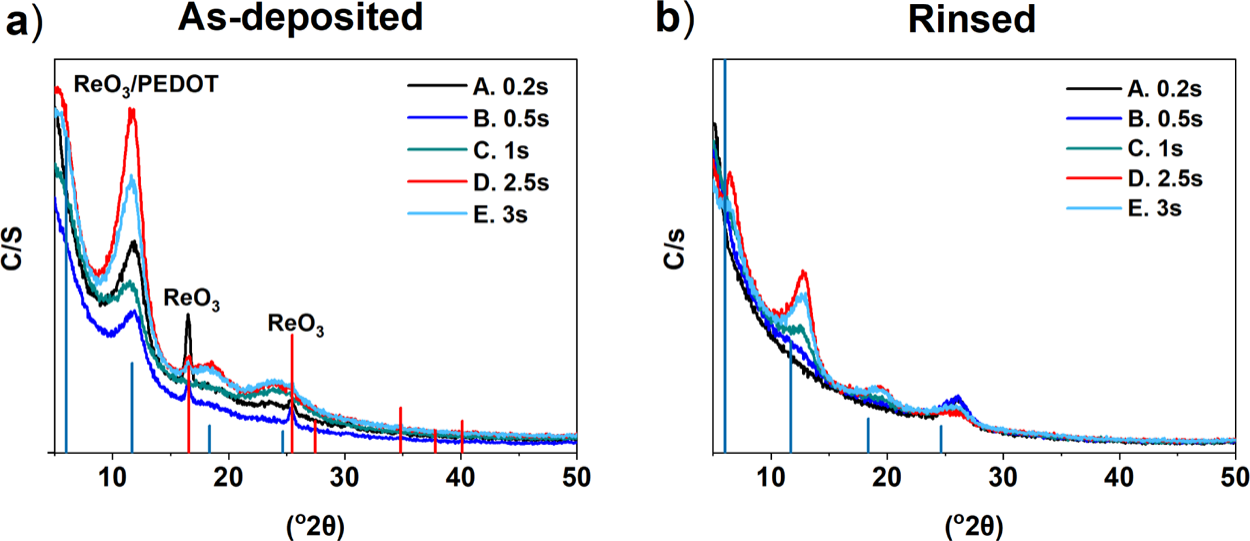
GIXRD reflection patterns of PEDOT films on Si. Films were deposited at 200 °C with 1000 cycles. (a) ReCl_5_ pulse, EDOT pulse, and purge durations were (0.2, 0.5, 1, 2.5, and 3 s), 2.0 s, and 2.0 s, respectively. (b) After the water rinsing. The bars indicate positions of reflections measured from the spin-casted PEDOT films (blue bars)^58^ and ReO_3_ (red bars).^59^

GIXRD studies on the PEDOT thin films were performed in order to identify the PEDOT structure as well as to identify the rhenium- and chlorine-containing particulates left on the film surface after the deposition. We compared our X-ray diffraction (XRD) results to those reported by Aasmundtveit et al.^58^ who studied PEDOT crystallinity with synchrotron radiation. It has been reported earlier^7^ that PEDOT has a paracrystalline structure with broad X-ray reflections without long-range order. This was seen also in all the diffraction patterns of our films, as broad reflections were observed that match with the PEDOT structure (Figure 8).

The as-deposited PEDOT films deposited with short ReCl_5_ precursor pulses of 0.2, 0.5, and 1.0 s additionally had reflections that matched with the ReO_3_ structure (Figure 7a). These reflections disappeared upon rinsing with water, which implies that ReO_3_ was in the particles that were removed by the rinsing (Figure 8b).

**Figure 9:**
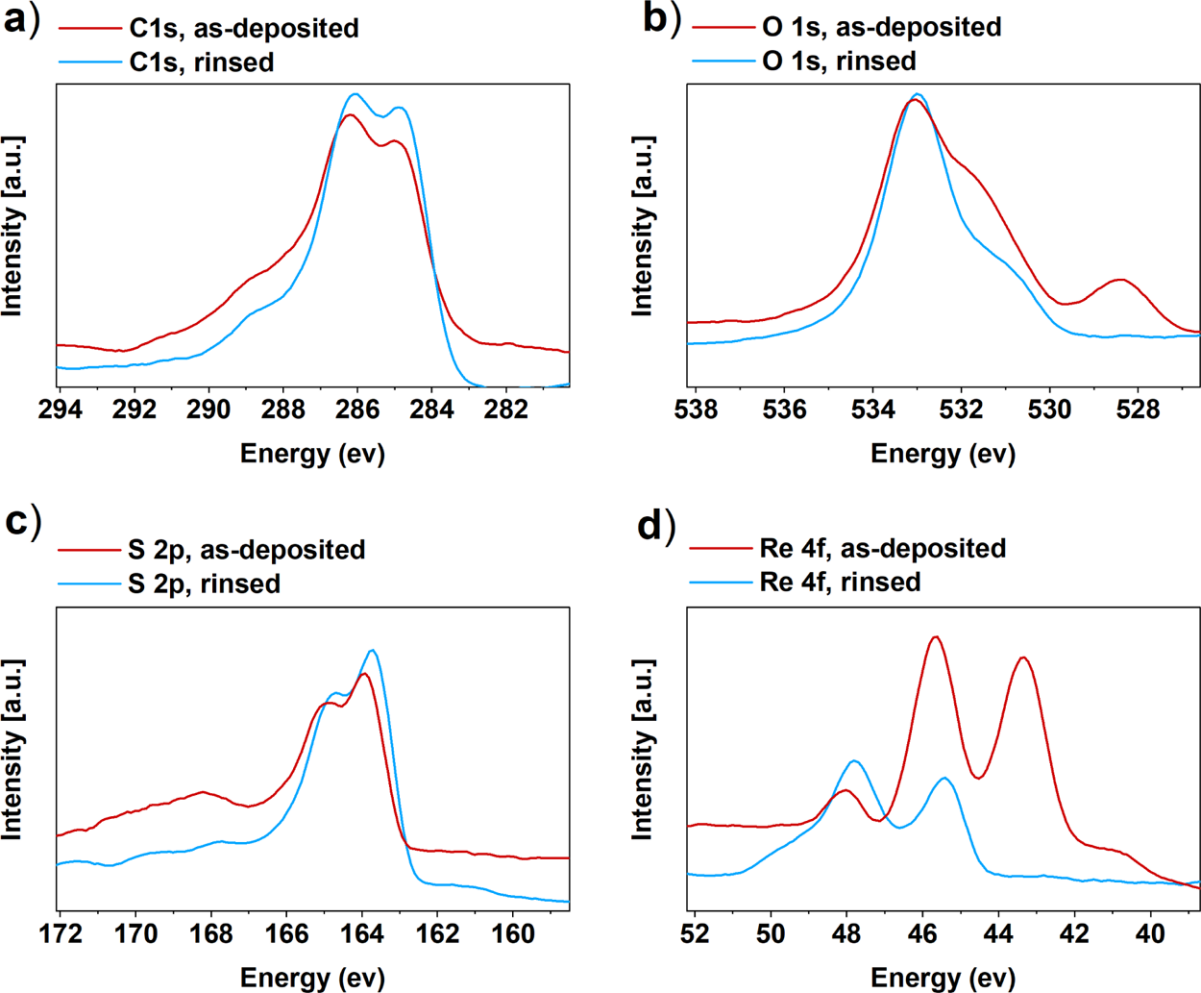
XPS results measured from the as-deposited and water-rinsed films. (a) Carbon 1s, (b) oxygen 1s, (c) rhenium 4f, and (d) sulfur 2p.

Detailed compositional studies with XPS confirmed the presence of carbon, sulfur, and oxygen. Chlorine was not detected in the as-deposited or water-rinsed PEDOT films but rhenium impurities in different oxidation states were detected. In the as-deposited films, there were 1.6 at. % Re and in the water-rinsed 0.5 at. %. These residual concentrations compare favorably with the 6 at. % Mo found from the PEDOT films deposited using MoCl_5_ as the oxidant.^37^Figure 9 represents the spectra of each element. The carbon 1s peak is a singlet and the expected peaks for PEDOT are at 285.0 and 286.1 eV positions in a 1:2 ratio,^16,60^ but instead the peak at 284.7–285 eV was more intense because of hydrocarbon contamination. Contaminants are probably the reason for the tails at 287–289 eV, which refer to carbonyl groups.

The main oxygen 1s peak at 533.6 eV is from PEDOT,^16,61^ and the shoulder at 531 eV is probably due to contamination (hydroxides). A special feature found in the as-deposited film is the peak at 528.3 eV that could be from rhenium oxide; that peak was absent in the spectrum of the water-rinsed sample. Sulfur 2p-doublet peaks at 163.5 eV (2p_3/2_) and 164.7 eV (2p_1/2_) are from PEDOT.^16,60^

Water rinsing decreased the Re content from 1.6 to 0.5 at. %. Although the large ReO_3_-containing particulates were removed by rinsing, some Re was still left in the films. Rhenium 4f-line is a doublet and it seems to consist of four different oxidation states (0, II, VI, and VII) for the as-deposited film, whereas for the water-rinsed film besides the IV and VII oxidation states, ReCl_*x*_ and ReOCl_*x*_ were identified. While no chlorine itself could be detected with XPS, the films could still contain such minor amounts of chlorine that affected the Re peak positions. Therefore, it was not trivial to fit the data to identify the oxidation state of rhenium in the chloride, oxide, and oxychloride compounds. Therefore, the curve fit was made for four components.^62^

**Figure 10:**
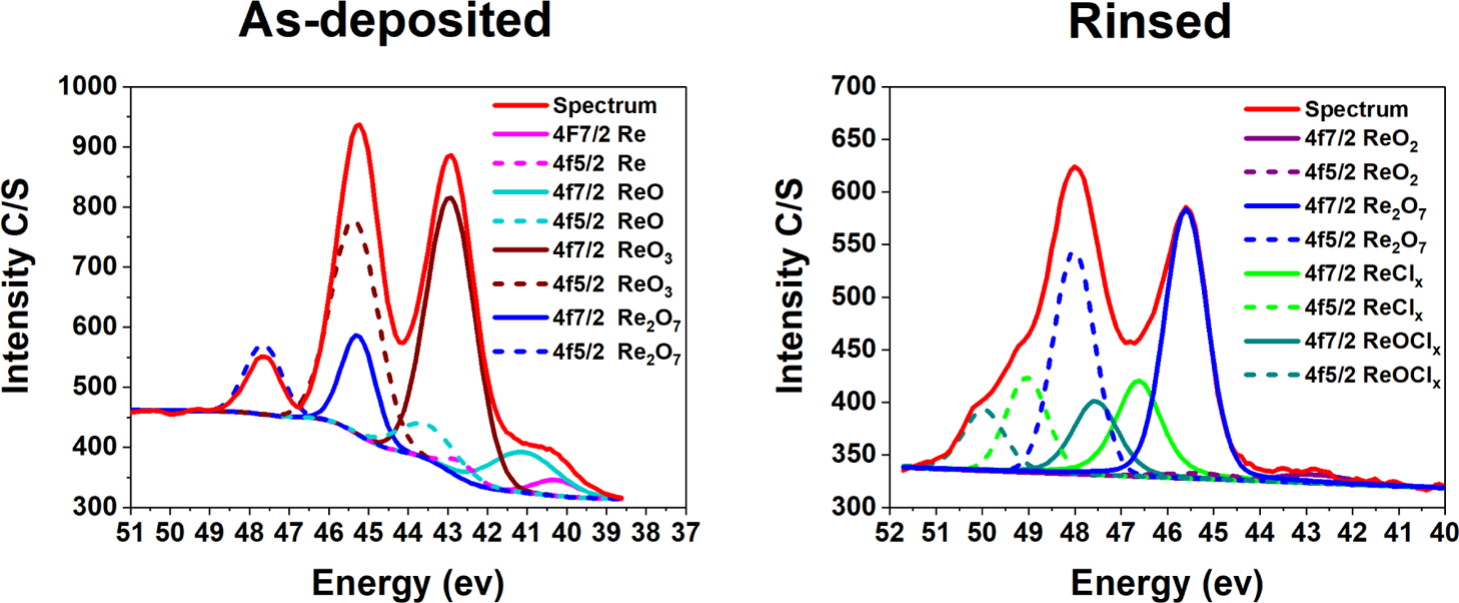
Re 4f curve fit for four oxidation states for the as-deposited and water-rinsed films.

From the relative intensities, one can calculate that rhenium detected in the as-deposited film (1.6 at. % in total) consisted of the following oxidation states: 3% metallic Re, 13% Re(II), 66% Re(VI), and 17% Re(VII). After the water rinsing, the distribution (0.5 at. % in total) was changed to 3% Re(IV), 57% Re(VII), 24% ReCl_*x*_, and 17% ReOCl_*x*_ (Figure 10). This further supports that the particulates on the as-deposited films consisted of ReO_3_. GIXRD showed reflections that were complying with rhenium in the VI oxidation state. No XRD reflections that would match with the metallic rhenium or rhenium(IV) compounds in the water-rinsed films were observed. The results indicate either the amorphous nature of the rhenium compounds in the zero and IV oxidation states or that the rhenium impurities are dissolved in the PEDOT.

**Figure 11:**
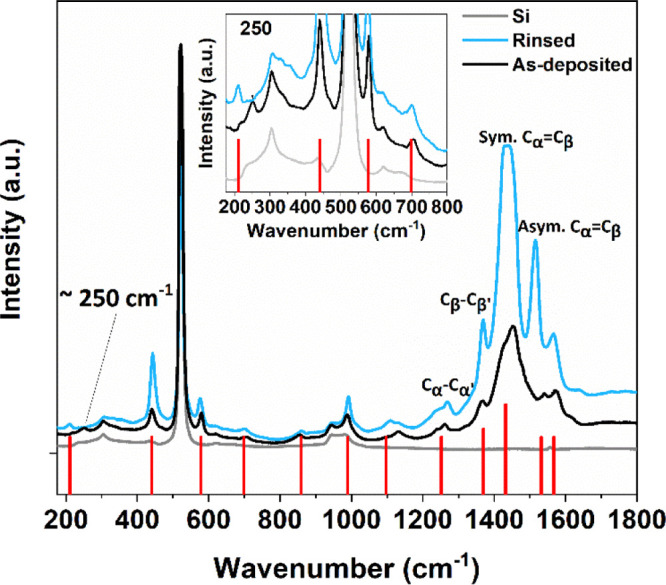
Raman spectra of the PEDOT thin films under green light excitation (λ = 532 nm). The red bars indicate the positions of Raman peaks of the PEDOT structure.

Raman scattering spectroscopy is also a very powerful tool for analyzing and chemical monitoring of PEDOT fingerprints.^63−65^Figure 11 represents the Raman spectra of the PEDOT films deposited with 1.0 and 2.0 s pulse times of ReCl_5_ and EDOT precursors, respectively.

**Scheme 2:**
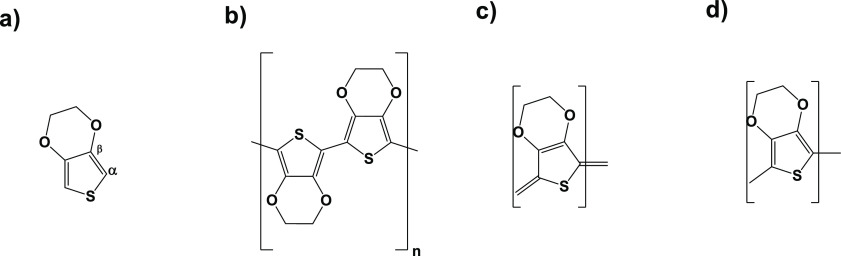
(a) EDOT, (b) PEDOT, (c) quinoid, and (d) benzoid structures in PEDOT

The Raman spectra confirmed the PEDOT structure. The vibrational modes of PEDOT are located at 1532, 1432, 1370, and 1252 cm^–1^, and assigned to the C_α_=C_β_ asymmetrical, C_α_=C_β_ symmetrical, C_β_–C_β′_ stretching, and C_α_–C_α′_ inter-ring stretching vibrations, respectively (see Scheme 2a for the carbon labelling).

PEDOT has two postulated resonance structures in its doped state, quinoid and benzoid forms as depicted in Scheme 2c,d.^66^ PEDOT in the benzoid form preserves its aromaticity through the C_α_–C_β_ bond via two conjugated π-electrons, whereas in the quinoid form there are no conjugated π-electrons on the C_α_–C_β_ bond and it has more planar structure compared to the benzoid form. The quinoid structure has more rigidity than the benzoid structure. The rigid quinoid structure enables strong interactions between the PEDOT chains and thereby high charge carrier mobility.^33^ Raman vibrations indicated the existence of both the rigid quinoid (linear conformation) and benzoid structure (coil conformation) in the doped state of PEDOT.^65^

EDOT monomer as a PEDOT building block also has a considerable effect on the rigid backbone structure of the polymer. The EDOT monomer has two oxygens in its structure and the oxygens play an important role in stabilizing the positive charges and radicals in the conjugated polymer chains.

According to the Raman spectra, PEDOT has an intermediate structure consisting of the C_α_=C_β_, (C_β_–C_β_, C_α_–C_α_) vibrational modes, which are attributed both to the benzoid and quinoid forms in the conjugated backbone of the polymer. There was also a weak mode at ca. 250 cm^–1^ in the as-deposited film that was not seen in the rinsed film. This weak vibrational peak is close to those of ReO_2_ (245 cm^–1^)^67^ and ReO_3_ (243 cm^–1^).^68^

## 3 Conclusions

The oMLD technique is a feasible way to deposit PEDOT thin films. PEDOT thin film deposition was carried out from EDOT and ReCl_5_. The films with the best qualities were obtained at a deposition temperature of 200 °C, where a growth rate of 0.6 Å cycle^–1^ was measured after water rinsing. After the deposition, there were rhenium and chlorine impurities in the film, but with a postdeposition water rinse, the majority of these impurities were removed. No chlorine was detected by EDS after the water rinse but there were some Re residuals detected by EDS and XPS for the both the as-deposited and water-rinsed films. The films were uniform, transparent, and showed a high electrical conductivity of 2000 S cm^–1^.

## 4 Materials and Methods

### 4.1 Film Deposition

The PEDOT thin film depositions were carried out in a hot-wall, flow type F120 ALD reactor (ASM Microchemistry Ltd.). The pressure inside the reactor was ca. 2 mbar. Monomeric EDOT (TCI, 95–98%) and the oxidants ReCl_5_ (Strem chemicals, IMC, 99.9%–Re), Br_2_ (ACROS organics, >90%, extra pure), SbCl_5_ (abcr chemicals, 99%), and VOCl_3_ (abcr chemicals, ≥99%) were used as received. Br_2_ and VOCl_3_ were introduced to the reactor from external containers because of their high volatility at room temperature. SbCl_5_ is also a highly volatile liquid and was evaporated from an open glass boat inside the reactor at 25–30 °C. EDOT was evaporated and ReCl_5_ was sublimed from open glass boats inside the reactor at temperatures of 35–45 and 110–115 °C, respectively. Nitrogen (N_2_, AGA, 99.999%) was used as a carrier and purging gas. The films were deposited on silicon pieces (5 × 5 cm^2^, native oxide) and soda-lime glass substrates. The as-deposited films were rinsed with deionized water at 25 °C.

### 4.2 Film Characterization

The morphology of the films was studied with a Hitachi S-4800 field emission scanning electron microscope. EDS measurements were performed with an Oxford INCA 350 energy spectrometer connected to the field emission scanning electron microscope. The crystallinity of PEDOT was studied with a Rigaku Smartlab X-ray diffractometer utilizing Cu Kα-radiation in the grazing incidence geometry (GIXRD, incident angle 1°).

Further compositional studies were performed with XPS (Phi Quantum2000) using monochromatized Al Kα-X-rays. The used spot size was 100 μm and the analyzer pass energy was 117.4 eV for the survey and depth profiles and 23.5 eV for the individual peaks.

A confocal Raman microscope (NT-MDT Ntegra) with a 532 nm laser and a 100× objective lens was used to measure the micro-Raman spectra in the backscattering geometry. The nominal output power of the laser was 20 mW. Laser exposure times were 15 s and the number of exposures was 40 for a single measurement.

Resistances of PEDOT thin films deposited on glass substrates were measured at room temperature using a four-point probe instrument (CPS Probe Station, Cascade Microtech combined with Keithley 2400). The as-deposited samples were stored in a desiccator prior to the measurement in order to minimize their exposure to the ambient air.

Film thicknesses were measured using a FS-1 ellipsometer (Film-Sense). Electrical conductivity of the films was calculated and compared for the both the as-deposited and water-rinsed films. The transmission spectra were measured using a UV–Vis spectrophotometer (Hitachi U-2000 Spectrophotometer) using bare soda-lime glass as the reference.
